# Gastrointestinal Disorders and Atopic Dermatitis in Infants in the First Year of Life According to ROME IV Criteria—A Possible Association with the Mode of Delivery and Early Life Nutrition

**DOI:** 10.3390/jcm13040927

**Published:** 2024-02-06

**Authors:** Maciej Ziętek, Małgorzata Szczuko, Tomasz Machałowski

**Affiliations:** 1 Department of Perinatology, Obstetrics and Gynecology Pomeranian Medical University in Szczecin, 70-204 Szczecin, Poland; maciejzietek@tlen.pl; 2 Department of Human Nutrition and Metabolomics, Pomeranian Medical University in Szczecin, 70-204 Szczecin, Poland; malgorzata.szczuko@pum.edu.pl

**Keywords:** cesarean section, functional gastrointestinal disorders, nutrition

## Abstract

**Background:** Functional gastrointestinal disorders are very common condition. The aim of this study is to evaluate the implications of the mode of pregnancy termination and early infant feeding on the incidence of gastrointestinal disorders and atopic dermatitis at birth and 3, 6, and 12 months of age. **Methods:** This study included 82 pregnant women and their newborns born at term. All newborns were examined at birth and 3, 6, and 12 months of age according to the ROME IV criteria. **Results:** In children born after cesarean section, the incidence of regurgitation was significantly higher. In children fed mostly or exclusively with formula, dry skin with allergic features was observed more often compared to breastfed children, but this relation was statistically significant only at the age of 12 months. The use of antibiotic therapy increased the risk of allergic skin lesions by almost seven times at 3 months of life. Gastrointestinal disorders in the form of regurgitation, colic, and constipation occur within the period of up to 12 months of the child’s life and may be related to the mode of the termination of pregnancy via cesarean section and the use of artificial feeding or antibiotic therapy. The occurrence of atopic dermatitis in infants at 12 months of life is correlated with the mode of the termination of pregnancy after cesarean section. **Conclusions:** One of the risk factors for the occurrence of atopic dermatitis and gastrointestinal disorders in the period up to 12 months of the child’s life may be a cesarean section and the use of formula feeding or antibiotic therapy.

## 1. Introduction

Functional gastrointestinal disorders (FGIDs) comprise a diverse combination of chronic or recurrent symptoms, the presence of which is not associated with the structural or biochemical abnormalities of a child. They occur with an incidence of 15–30%, and their severity and nature depend on a number of factors, including age and physiological, autonomic, emotional, and intellectual development [1,2,3]. The symptoms of gastrointestinal disorders are most commonly caused by the immaturity of the gastrointestinal tract, the nervous system, and abnormalities in the gut microbiome of infants. FGIDs may occur in normally developing children or be a consequence of abnormal behavioral responses to internal or external stimuli. Despite the precise classification of the above disorders within the framework of the Rome IV criteria [4], their pathophysiology and importance for the further development of the child are still poorly understood. We should, therefore, treat FGIDs as disorders of the brain–gut axis, for just as the central nervous system influences the functioning of the gut, the gut exerts an influence on the brain [5,6,7]. In regulating gut–brain interactions, neurogenic, endocrine, and immunological mechanisms are involved, which may be modified by the gut microbiota intestinal microbiota. The microbiome, through secreted metabolites, can exert both salutary and harmful effects on the intestinal mucosa. Factors disrupting normal gut colonization, leading to dysbiosis, can have a significant impact on the occurrence of FGIDs. Since the association of FGIDs, according to the Rome IV Criteria, with the mode of delivery and child feeding has not been studied and published before, in this work, we conducted such an analysis, which is a novel scientific endeavor. The occurrence of FGID is not an indication to discontinue breastfeeding, which should, in fact, be actively encouraged. In infants fed modified milk, special formulas may be considered if reassurance and nutritional recommendations based on adequate milk volume and frequency do not lead to sufficient improvement. In cases of the absence of organic disease, any pharmacologic intervention is unlikely to be helpful or effective. Furthermore, medications may cause side effects and unnecessarily expose the child to additional stress. The occurrence of FGIDs often leads to a vicious circle: the newborn’s symptoms make the parents anxious, which leads to the implementation of drugs available at the pharmacy without consulting a doctor. Consequently, the symptoms persist, and diagnosis is delayed. Environmental factors and the type of food consumed by the infant in the early postnatal period play a significant role in the development of the infant’s immune system, and it is suspected that they may also be involved in the development of not only FGIDs but also atopic diseases in children. There is also medical evidence that there may be a link between FGIDs and the way pregnancy is terminated. Numerous epidemiological studies have shown an association between cesarean section and an increased risk of developing immunological diseases, including early and persistent wheezing and bronchial asthma [4,8,9], allergic rhinitis, ulcerative colitis, type 1 diabetes, celiac disease, being overweight, and obesity [4,10]. The disturbed composition of the neonatal gut microbiome, resulting from limited contact with maternal rectal and vaginal flora and leading to dysbiosis, may be an initiating factor in the onset of FGIDs. Given that there is an association between the early composition of the neonatal gut microbiota and the occurrence of chronic diseases later in childhood, it is reasonable to assume that impaired intestinal colonization due to cesarean delivery may play an important role in the etiology and pathogenesis of childhood FGIDs. Some symptoms of FGIDs, such as colic, show an association with the presence of more colonies of proteobacteria, including Escherichia, Klebsiella, and Pseudomonas, while Firmicutes and Actinobacteria are more prevalent in infants without colic [11]. The modification of the composition and activity of the child’s intestinal microbiota through dietary interventions using probiotics may be one of the directions for the prevention and treatment of infants with FGIDs and allergic diseases. It has been proven that selected probiotic strains from the genus Lactobacillus can alleviate IBS symptoms. The relative risk (RR) value for symptom improvement was 7.69, which means that clinical improvement is more than seven times more likely in those taking the probiotic compared to those not taking such supplementation [12,13,14]. The gut microbiota occupies a prominent position in the structure and function of the brain–gut axis, which is a bidirectional pathway for the exchange of neural and biochemical signals between the central nervous system and the gastrointestinal tract. The intestinal barrier, which includes the microbiota, is, therefore, a point of interest for potential therapeutic interventions in FGIDs [13,14,15,16].

### Objectives

The aim of this study is to evaluate the implications of the mode of pregnancy termination and early infant feeding on the incidence of gastrointestinal disorders and atopic dermatitis at birth and 3, 6, and 12 months of age, according to the ROME IV criteria.

## 2. Material and Methods

This was a prospective cross-sectional study with a group of similar patients that had a regular follow-up at the same time point. The study included 82 randomly selected pregnant women and their newborns born near the term of gestation at the Department of Perinatology, Obstetrics, and Gynecology in Szczecin, Poland. The data were collected between October 2020 and September 2022. The study group was randomly selected from a population list of woman–infant pairs, meaning that random chance decided which woman–infant pair entered the sample. A simple draw (non-return) type of random selection was used for this purpose. The inclusion criteria included a pregnancy terminated between 37 + 0 and 41 + 6 weeks of a singleton pregnancy, maternal consent for the study, the physiological course of the pregnancy and childbirth, a minimum of 8 points with respect to the 5-min Apgar score, and no developmental birth defects. The exclusion criteria included pregnancies terminated before 37 weeks of gestation, comorbidities complicating pregnancy, severe birth status of the newborn, multifetal pregnancy, and a lack of written maternal consent for the study. In pregnant women, anthropometric data, the method of termination of pregnancy, and other medical data, including the course of the current pregnancy and family history of allergy, were considered (Table 1). A Detecto PD200 medical scale (DETECTO Cardinal Scale Manufacturing Co., 203 E. Daugherty, Webb City, MO 64870, USA) has been used for the digital weight, height, and BMI measurements. All infants were examined by a pediatrician at birth and at 3, 6, and 12 months of age consecutively (Table 2). The neonatal clinical examination of the babies was performed at home in their place of residence by a specialist physician. The same doctor carried out examinations during all visits, as it helps the avoidance of methodological errors and differences in diagnoses, especially, since the doctor has many years of experience. At each medical visit, a functional gastrointestinal disorders questionnaire according to the Rome IV: The Rome IV Diagnostic Questionnaire for Functional Gastrointestinal Disorders (R4PDQ—A parent report form for neonates and toddlers 0–3 years old) was completed, and data were collected with respect to the presence of atopic dermatitis and how the children were fed (breastfeeding, formula feeding, supplemental feeding, and supplemental feeding with probiotics). Atopic dermatitis was diagnosed on the basis of clinical signs such as the following: a rash with eczema on the cheeks, forehead, or scalp, which was associated with dry and itchy patches of skin. In some cases, the lesions spread to the knees, elbows, and trunk. The experts on FGIDs and parents reviewed the questionnaire for content, understandability, and completeness. The content’s validity was established by comparing the neonatologist and questionnaire diagnoses in the studied group of children. The validation of the questionnaire met the guidelines on how to translate and validate questionnaires, which are included in the document defining the principles of translation and its adaptation to the conditions of the Polish Caucasian population. The high repeatability of the results obtained with the use of the R4PDQ questionnaire allows us to consider the evaluated questionnaire as an accurate measurement tool that may be a source of reliable information with respect to the clinical symptoms of intestinal disorders. In the diagnosis of the functional disorders of the gastrointestinal tract, data obtained from the history and physical examination are of great importance, taking into account alarm signals, which include chronic pain in the right upper or right lower quadrant; pain when waking the child from sleep; swallowing disorders; prolonged vomiting; gastrointestinal bleeding; diarrhea occurring at night; positive family history of inflammatory bowel disease, coeliac disease, or peptic ulcer disease; arthralgia; perianal lesions; weight loss; slowed growth rate; and unexplained fevers. During the examination, weight, height, and head and abdomen circumferences were recorded, and centile grids of the Polish population, according to the WHO’s growth standards for children aged 0–3 years, were used [8]. During this period of life, children cannot voice complaints, such as nausea or pain. Therefore, the clinician must rely on the parents’ interpretations, which, combined with his knowledge, skills, and experience, allow him to differentiate between health and disease in the first instance.

Statistical analysis was performed using STATA 11 statistical software (StataCorp LLC, 4905 Lakeway Drive, College Station, TX 77845-4512, USA), license number 30110532736. The Kolmogorov–Smirnov test for normality of distributions was used. If *p* < 0.05, then the Mann–Whitney U or Kruskal–Wallis test was used. For normal distributions, the Student’s *t*-test or ANOVA was used. Correlations were calculated using Spearman’s rank test.

## 3. Results

The study included 82 randomly selected pregnant women and their newborns born near the term of gestation at the Department of Perinatology, Obstetrics, and Gynecology in Szczecin, Poland. Of the infants in the study, 42% were born from nulligravidae. An allergy history was found in 26% of the mothers in the study. Allergies relative to grass pollen (23%) and house dust mites (19%) were the most common. In the fathers of the examined infants, 29% were allergic, and the most common allergen in this group was grass pollen (62%). The median APGAR score was 9.0 (8.0–10.0 IQR) at the fifth minute.

### 3.1. Functional Gastrointestinal Disorders

**Figure 1:**
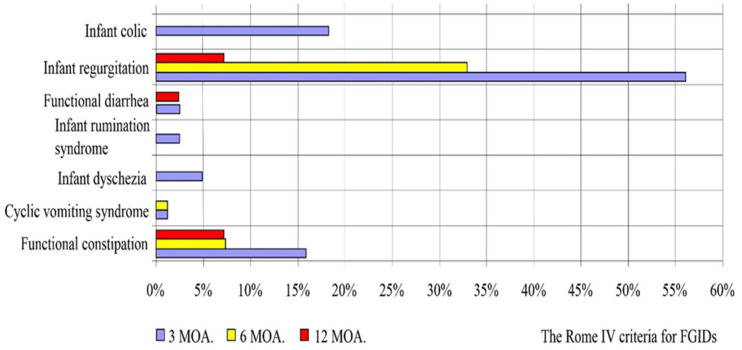
Functional gastrointestinal disorders according to Rome IV criteria.

**Figure 2:**
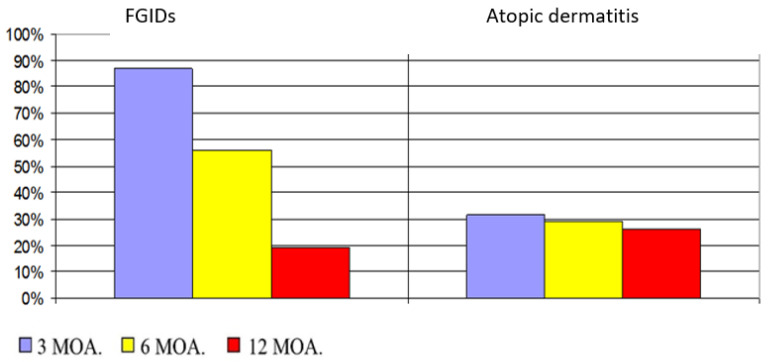
Incidence of gastrointestinal disorders (FGIDs) and atopic dermatitis in infants at 3, 6, and 12 months of age.

In all infants, the process of postpartum adaptation was normal during the first 2 days of life. All infants were discharged home in good condition and breastfed. On the basis of clinical examination and the diagnostics of functional gastrointestinal disorders according to the Rome IV criteria carried out during 3, 6, and 12 months of life, differences in the occurrence of the particular examined parameters were demonstrated (Figure 1). At 3 months of life, the most frequently observed functional gastrointestinal disorder was neonatal regurgitation (56.10%), which gradually decreased to 32.93% and 7.14% at 6 and 12 months of life, respectively. The second most frequently observed symptom was infantile colic, which, although present in 18.29% of infants at 3 months of age, did not appear thereafter. Constipation was also observed in all studied infants, which occurred in 15.85%, 7.32%, and 7.14% consecutively at 3, 6, and 12 months of life (Figure 1). Considering the cumulative incidence of gastrointestinal disorders, it was observed that the most frequent gastrointestinal disorders occurred at 3 months of life in 86.59% of infants, gradually decreasing to 56.10% and 19.05% at 6 and 12 months of life, and these differences were statistically significant (*p* < 0.00001) (Figure 2).

### 3.2. Atopic Dermatitis

Symptoms of atopic dermatitis were observed in 31.71%, 29.27%, and 26.19% of the infants at 3, 6, and 12 months of life, respectively, and these differences were not statistically significant. However, a statistically significant positive correlation (*p* = 0.0042; r = 0.31) between the occurrence of gastrointestinal disorders and allergy at 3 months of life was found. Atopic dermatitis was more frequent in female newborns, and this correlation was statistically significant (*p* = 0.048). No similar correlation was found at 6 and 12 months of life (Figure 2).

### 3.3. Mode of Delivery

**Figure 3:**
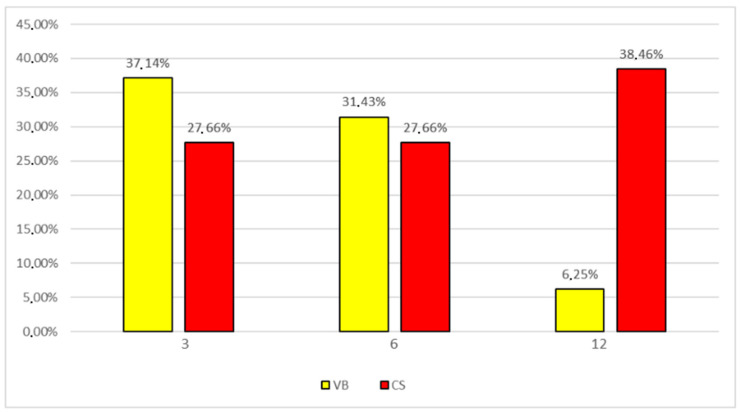
Incidence of atopic dermatitis in infants at 3, 6, and 12 months of age depending on the mode of delivery: vaginal birth (VB) and cesarean section (CS).

The analysis of the mode of delivery revealed that cesarean section is associated with a higher incidence of allergy in infants at the age of 12 months (38.46% vs. 6.25%), and this relation was statistically significant (*p* < 0.005) (Figure 3). The overall risk of allergy at 12 months of life was over nine times higher in children born after cesarean section (OD 9.37; *p* = 0.044). There was no significant increase in allergy in 3 and 6 months of life in infants born after cesarean section. The surgical termination of pregnancy via cesarean section did not show a significant association with most gastrointestinal disorders in infants in any of the analyzed periods of neonatal studies. Only in the group of children born after a cesarean section was regurgitation more frequent, and this relation was statistically significant (*p* = 0.0434). Infants with normal postpartum adaptation in the majority of cases (80%) did not show any gastrointestinal disorders at a later stage. In 20% of infants with normal adaptation, bloating, gas, and colic were observed. However, this relation was statistically significant (*p* = 0.00544) only in the third month of life. The Spearman’s rank correlation test showed that the occurrence of both gastrointestinal disorders and allergies significantly correlated with gender only at 6 months of life (*p* = 0.0019). There was no correlation between the birth weight and the occurrence of gastrointestinal disorders and atopic dermatitis; however, a significant correlation was found for pregnant women’s body weights and BMIs, but only at 12 months of age.

### 3.4. Nutrition

**Figure 4:**
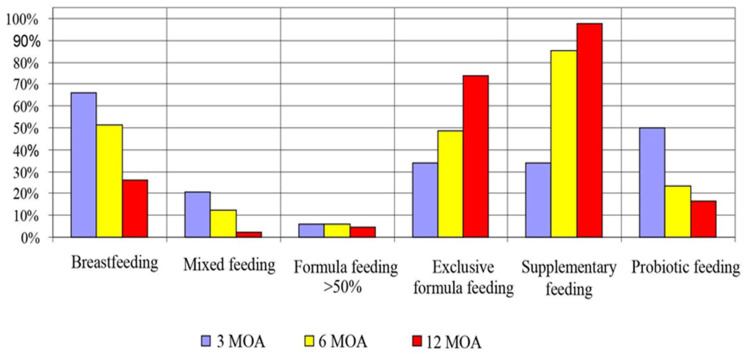
Infants feeding at 3, 6, and 12 months of age.

According to the study, the majority of infants were breastfed at 3 months of life (65.85%), and with the passage of time, their percentage was gradually reduced at 6 and 12 months of life, respectively, to 51.22% and 26.19% (Figure 4). This relationship is statistically significant. The use of probiotics in the diet of newborns showed a similar trend. The highest number of infants received probiotic supplementation at 3 months of life (50.00%) and, respectively, at 6 and 12 months of life, 23.17% and 16.67%. As the infant grew older, an expansion of the diet to include solid foods and an increasing use of various types of baby formula milk at the expense of natural breastfeeding were observed. On the other hand, with the passage of time, a more frequent introduction of nutritional supplementation was observed at the expense of natural feeding. The results of the study showed that, at 12 months of age, 73.81% of infants with a recommended properly expanded diet of solid foods in place of maternal milk were fed exclusively with formula nutrition (Figure 4). In order to check which variables influence gastrointestinal complaints and skin lesions, a multivariate discriminant analysis was performed. It was shown that neonatal constipation was more frequently observed when a probiotic was introduced into the diet, and this relationship was statistically significant (*p* = 0.0045). When both the time function of using a given feeding method and the probiotic were eliminated, it was shown that bloating and gas were more common in breastfed babies (*p* = 0.0490). In this group, the symptoms of atopic dermatitis and rash were also more frequent. In more than 50% of artificially fed children and only artificially fed children, dry skin with allergic features was observed more often, but this relationship was statistically significant only at 12 months of age. The presence of colic tended to be more common in infants who were treated with the probiotic and those who were fed formula, but this relationship was at the border of statistical significance. A logistic regression model was used to estimate the risk of various digestive ailments and skin allergic symptoms. The use of a probiotic increases the risk of constipation in the third month of life (*p* = 0.045) fourfold. On the other hand, bloating and gas occur almost four times more often in the third month of life when the child is artificially fed or formula-fed. When a child is fed formula, the overall risk of PP complaints increases over six times more often at 12 months of age (*p* = 0.028). The use of antibiotic therapy increases the risk of skin lesions of an allergic nature during 3 months of life by almost seven times.

## 4. Discussion

FGIDs are common in newborns and infants and concern about 26–60% of them [8,9,10]. Our study found a statistically significant phenomenon, decreasing with the age of the infant: the frequency of disorders in the FGID spectrum. The frequency of disorders at 3 months of age was 86.59%, 6–56.1% at 6 months of age, and 19.05% at 12 months of age. This phenomenon may be influenced by the adaptation process of the infant to the environment, particularly by developing tolerance to food allergens, increasing the maturity of neurological and digestive systems and the brain–gut axis that underlies the disorders [17]. In the presented study, the most common symptoms of FGIDs were regurgitation at 56.1% during the third month of life, with a subsequent downward trend—32.9% in the sixth month and 7.14% in the twelfth month of life. Similarly, regurgitation, as the most common symptom in the FGID spectrum, is reported to be present in 40% [18,19] and 30% of infants [20]. Kee Seang Chew et al. found regurgitation to appear in only 10.5% of subjects, but the study was conducted in Asia, where the overall frequency of FGID symptoms is low, occurring in 14.6% of infants [21]. Intestinal colic is indicated in many studies as the most common disorder from the FGID spectrum, where it affects up to 57.6% of infants with FGIDs [4]. In our study, it was ranked second and concerned 18.28% of infants. Colic occurs only at 3 months of age, which is typical for this disorder and results from the diagnostic criteria. According to the Rome IV criteria, this disorder appears and disappears by the fifth month of life, and if the parents’ care is conscientious and constant, functional gastrointestinal disorders do not pose a danger to the child [18]. The third most common symptom—constipation—occurred in all newborns, with 15.85% of infants at 3 months of age; then, its incidence decreased with the infant’s age. These results are consistent with many analyses [18,20]. However, Chogle et al., while studying the infant population in Colombia, assessed constipation as the most common FGID disorder, with a frequency of 16.1% [11]. The incidence of atopic dermatitis did not differ significantly depending on the age of the infant, ranging from 26 to 31%. There is a general upward trend in the incidence of skin allergies worldwide. Symptoms most often appear from early infancy to the age of 1. It is believed that environmental factors trigger an effect in neonates with a genetic predisposition [22]. Our study found a positive correlation between FGID symptoms and the occurrence of allergies, but only at 3 months of age. The source of this phenomenon may be a common etiological factor when the immunologically immature organism reacts to the allergen with both skin and gastrointestinal symptoms. The appearance of the above-described symptoms prompts parents to look for and exclude potential allergens, e.g., a nursing mother’s elimination diet, change from natural to artificial feeding, change in the type of milk formula, introduction of probiotics, and change in care cosmetics. Effectively eliminating the allergen could have contributed to the lack of persistence of the correlation between skin lesions and gastrointestinal symptoms after 3 months of age. The development of immunological tolerance, along with the exposure time, may also play an important role [23]. FGIDs are considered to be transient, self-limiting disorders that disappear with the infant’s age due to the maturation of the physiological mechanisms responsible for their formation [17,24]. It could also have contributed to the lack of persistence of the positive correlation between FGID and allergies after 3 months of age. The correlation between allergic disorders and FGID was more common in girls. The results vary from study to study. In Campeotto’s study, the above symptoms mainly occurred in boys [18]. In our study, a positive correlation was observed between the occurrence of colic and constipation (the latter only at 3 months of age) and the supply of a probiotic. One of the postulated etiological factors of colic is the dysbiosis of the intestinal flora. The supply of Lactobacillus reuteri to breastfed infants with symptoms of colic has proven therapeutic efficacy [12,25]. The correlation obtained may result from the fact that in the event of the appearance of FGID symptoms, probiotic therapy is implemented. Gastrointestinal symptoms, such as bloating and gas, were more frequently observed in breastfed newborns and infants. Allergic dermatitis and rash were also more common. Similar results were obtained in the Danish study [22]. The diet may have influenced this correlation. Formula-fed babies were given a constant formula, while breastfed babies could have been exposed to a variety of ingredients, including allergens, depending on the mother’s diet. Currently, elimination diets are not recommended for mothers who are breastfeeding healthy infants; therefore, they could also contain gas or products that are considered to be allergenic. Exclusive breastfeeding until the age of 3–4 months and mainly breastfeeding until the age of 6 months is of prophylactic importance in the development of allergies [26,27]. The described mechanisms include a passive mechanism—reducing the exposure to exogenous allergens—and an active mechanism—secretion into the milk of substances protecting against infections, stimulating the maturation of intestinal mucosa, stimulating the development of normal intestinal flora, and secreting a number of anti-inflammatory and immunomodulatory factors [28,29]. In our study, feeding with a milk mixture was associated with a 6-fold increase in gastrointestinal complaints; moreover, when only formula is used, or formula is used in >50% of feeding, skin allergies were observed, but only at 12 months of age. The fact that the percentage of naturally fed children decreased with age (3 months at 65.85% and 12 months at 26.19%) may account for the statistical significance in the appearance of allergy symptoms at 12 months of age. Moreover, after 6 months of age, expanding the child’s diet with solid foods is recommended. Some of them could have an allergenic effect, and in the absence of the protective role of breast milk, skin symptoms could be more strongly expressed with formula feeding. In the present study, the termination of pregnancy via cesarean section was nine times more likely to increase the risk of skin allergy at 12 months of age. Moreover, the need for antibiotic therapy was associated with a 7-fold risk of allergic skin lesions. The adaptation and development of the immune system are undoubtedly influenced by the intestinal flora of newborns. It differs depending on the pregnancy termination method. After a vaginal birth, the intestinal flora of newborns comprises more bacteria from the mother’s intestinal and vaginal flora; after a cesarean section, it comprises skin bacteria. Newborns born after cesarean section have a later maturation of the intestinal flora and a different cytokine profile [30,31,32,33]. Selma-Royo et al. found a stronger epithelial barrier function, a higher immune response associated with TL4 pathway activation, and the production of proinflammatory cytokines in neonates born after cesarean section [33,34]. Thus, the mode of delivery seems to be an important factor in the development of the immune system and, consequently, the occurrence of allergies. The implementation of antibiotic therapy in the mother, both in the perinatal period (as prophylaxis)—when the pregnancy is terminated via cesarean section—and in the infant at different stages of life significantly disrupts the composition of the intestinal flora, the function of enterocytes, and the activity of the local immune system, which may have contributed to an increased risk of skin allergies. However, studies are not consistent; Maeda et al. did not find a higher incidence of skin lesions in infants after cesarean section deliveries [30]. The presented study has limitations. It should be taken into account that the diagnosis of FGIDs, based on the Rome IV criteria, took place on the basis of an interview carried out with parents [28]. Their perception of the infant’s gastrointestinal physiology, emotional attitude, personal experiences, character traits, and family situation may have reduced the objectivity of the opinion. Some reports emphasize the higher incidence of FGIDs in children or firstborns and with divorced parents [15]. In this study, these factors were not investigated.

## 5. Limitations of the Study

This study has several limitations. Firstly, our results are based on the diagnosis of FGID symptoms in children, which was carried out in an interview with the parents; despite the use of an objective questionnaire, subjective information arose from the parents’ emotional attitudes and experiences. We cannot exclude inconsistencies and missed data. Secondly, the study group only comprised 82 pregnant women and their newborns, and some conclusions may not be confirmed in such a group of people, but the statistical significance of the obtained results encourages carrying out the above analysis in a larger group of women and their children. Third, the group is homogeneous; thus, the results can only refer to this population, and conclusions cannot be drawn about the entire population.

## 6. Conclusions

Summarizing, regurgitation, infant colic, and functional constipation are common FGIDs that often contribute to a visit to physicians during the first 3 months of an infant’s life. Atopic dermatitis and gastrointestinal tract disorders may be associated with a common mechanism triggering these reactions. One of the risk factors for the occurrence of these symptoms in the period up to 12 months of a child’s life may be the termination of pregnancy via cesarean section, the use of formula feeding, or antibiotic therapy.

The above observations have proven to be an important clinical application for family doctors, pediatricians, and GPs because they show the probable relationship between the occurrence of gastroenterological disorders, the method of delivery, and the overuse of antibiotics and formula feeding. The results are important, especially in the field of obstetrics in the Polish population, where the percentage of cesarean sections in 2022 was 48%, and in the previous year (2023), it exceeded 50% (according to WHO, this percentage should be about 15%). If the results of this work reach a wider population, perhaps the rate of elective cesarean sections could be reduced. The above relationship concerns the overuse of antibiotics in the Polish population, forcing doctors to prescribe them during viral infections, and the rapid abandonment of breastfeeding among patients after childbirth (exclusive breastfeeding reached 30% in the fourth month and in the sixth month, it went from 4 to 14% depending on the study). Patient awareness is the basis for improving population health.

## Figures and Tables

**Table 1:** Anthropometric data of the studied pregnant women.

Gestation/Delivery (*n* = 82)	Mean	SD
Age (years)	31.22	4.49
Body weight before pregnancy (kg)	68.28	17.59
Birth weight during delivery (kg)	82.20	16.55
BMI before pregnancy	24.56	6.05
BMI during delivery	29.57	5.62
Weight gain (kg)	14.24	7.01
Week of gestation completion	38.44	1.30
Natural childbirth *n* (%)	35 (42.7)	-
Cesarean section *n* (%)	47 (57.3)	-

**Table 2:** Anthropometric data of infants on the day of birth and at 3, 6, and 12 months of age.

Parameters	Mean	SD
Male *n* (%)	38 (46.3)	-
Female *n* (%)	44 (53.7)	
Neonatal birth weight (g)	3286	395
Neonatal birth weight (g) at 3 MOA (*n* = 82)	6340	651
Neonatal weight (g) at 6 MOA (*n* = 82)	8020	900
Neonatal weight (g) at 12 MOA (*n* = 42)	10,560	1040

MOA—months of age.

## Data Availability

The data presented in this study are availabe on request from the corresponding author (Tomasz Machałowski).
